# Pneumothorax and subcutaneous emphysema secondary to blunt chest injury

**DOI:** 10.1186/1865-1380-4-10

**Published:** 2011-03-21

**Authors:** Jahan Porhomayon, Ralph Doerr

**Affiliations:** 1 VA Western New York Healthcare System, Division of Critical Care and Pain Medicine, Department of Anesthesiology, State University of New York at Buffalo School of Medicine and Biomedical Sciences, VA Medical Center, Rm 203C, 3495 Bailey Ave, 14215, Buffalo, New York, USA; 2 Via Health of New York Health Care System, Rochester, New York, USA; State University of New York at Buffalo School of Medicine and Biomedical Sciences, 3495 Bailey Ave, 14215 Buffalo, New York, USA

## Abstract

This is the case of a patient with a history of blunt chest trauma associated with subcutaneous emphysema and pneumothorax. The patient complained of inspiratory stridor on presentation. Anatomical relationships can explain the pathophysiological process.

## Case report

A 49-year-old male presented to the trauma service 10 h after blunt chest injury. Initial presentation included respiratory failure with a respiratory rate of 26 beats per minute, a pulse rate of 110 beats per minute, and blood pressure of 150/80 mmHg. He complained of dysphonia and facial swelling.

**Figure 1:**
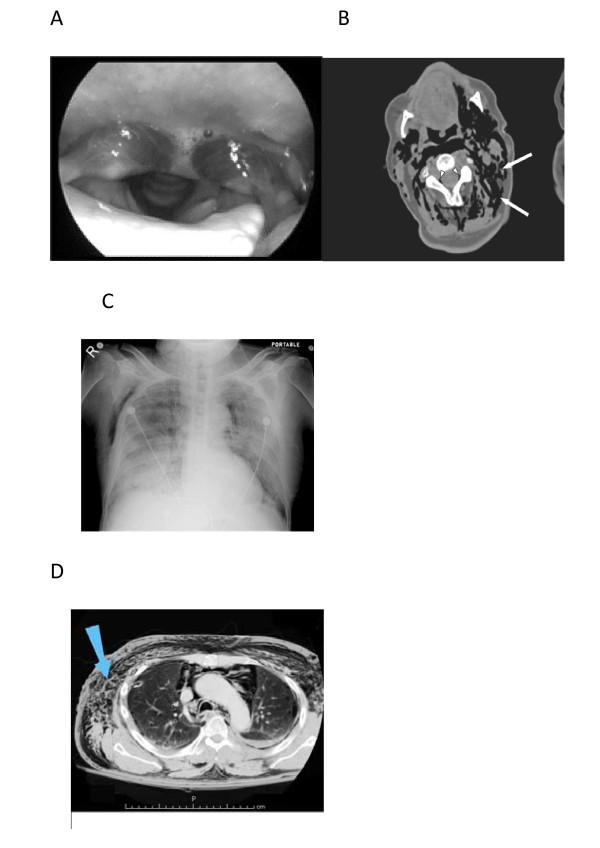
**Subcuatneous and Submucosal emphysema. A)**Pharyngeal submucosal emphysema **B) **CT image with subcutaneous air** C) **The chest radiograph shows extensive subcutaneous emphysema **D) **The CT-scan  shows pneumothorax , pneumomediastinum and   subcutaneous emphysema

Physical examination revealed inspiratory dyspnea and crepitations suggestive of subcutaneous emphysema of the face, neck, and upper portion of his chest. Pharyngeal examination revealed swollen mucosa with crepitations on palpation (Figure 1a). Chest X-ray indicated extensive subcutaneous emphysema apparent in part as a group of muscles in the upper chest wall, but with no obvious pneumothorax (Figure 1c). Computed tomography of the chest confirmed subcutaneous and submucosal emphysema involving the pharynx. It also revealed obvious pneumomediastinum associated with left pneumothorax from rib fractures (Figure 1b and 1d). Physical examination and bronchoscopy ruled out laryngotracheal mucosal rupture. The patient remained dyspneic after placement of a chest tube. Twenty-four hours later, inspiratory dyspnea, dysphonia, and submucosal emphysema had resolved. Subcutaneous emphysema resolved in 4 days. The patient's recovery was uneventful.

## Discussion

**Figure 2:**
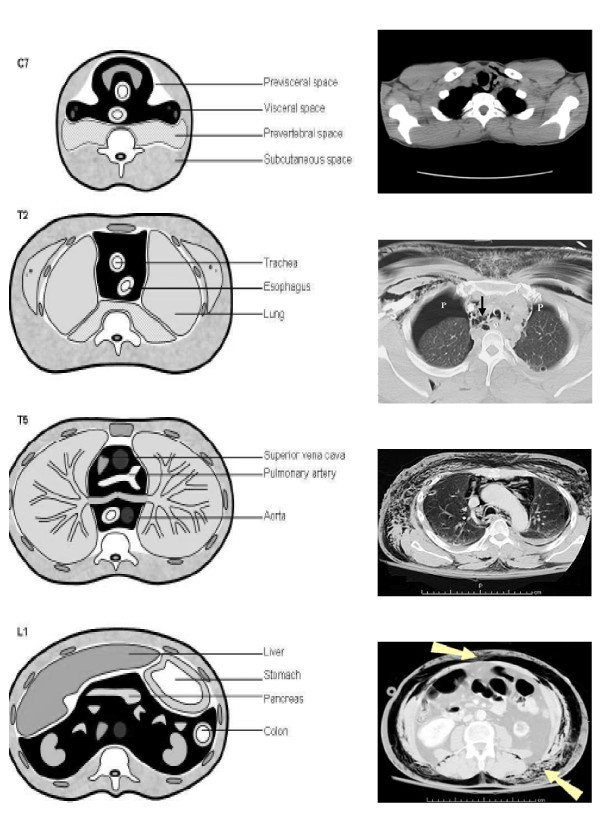
**Anatomical relationship of abdominal, thoracic and cervical fascial planes. A) **Anatomical relationship of  the cervical and thoraco-abdominal region** B) **Air can diffuse through cervical, mediastinal and retroperitoneal region

Subcutaneous emphysema can occur in critically ill patients after blunt trauma to the chest and result in a pressure gradient between the intra-alveolar and perivascular interstitial space [1,2]. The chest radiograph cannot exclude pneumothorax or pneumomediastinum. A CT scan is often needed for assessment of these conditions. Oropharyngeal subcutaneous emphysema has been described with dental surgery or spontaneous rupture of oropharyngeal or bronchial mucosa [3,4]. The association of submucosal emphysema with pneumothorax is rare. However, anatomical correlation among fascial planes of the cervical area, mediastinum, and retroperitoneum can explain this relationship [1] (Figure 2a and 2b).

## Competing interests

The authors do not have any financial and personal relationships with other people or organizations that could inappropriately influence (bias) their work. Examples of potential conflicts of interest include employment, consultancies, stock ownership, honoraria, paid expert testimony, patent applications/registrations, and grants or other funding.

## Authors' contributions

Both authors contributed to writing the manuscript.
